# Overview of Infectious Disease Surveillance System in Japan, 1999-2005

**DOI:** 10.2188/jea.17.S3

**Published:** 2008-01-30

**Authors:** Kiyosu Taniguchi, Shuji Hashimoto, Miyuki Kawado, Yoshitaka Murakami, Michiko Izumida, Akiko Ohta, Yuki Tada, Mika Shigematsu, Yoshinori Yasui, Masaki Nagai

**Affiliations:** 1 Infectious Disease Surveillance Center, National Institute of Infectious Diseases.; 2 Department of Hygiene, Fujita Health University School of Medicine.; 3 Department of Health Science, Shiga University of Medical Science.; 4 Department of Public Health, Saitama Medical University Faculty of Medicine.

**Keywords:** Sentinel Surveillance, Communicable Disease, Disease Notification

## Abstract

**BACKGROUND:**

In 1999 the Communicable Disease Prevention Law of Japan was completely revised into the "New" Infectious Disease Control Law, which reiterated the importance of surveillance and information dissemination and re-organized the surveillance system. This paper is an attempt to illustrate the potential impact of the new surveillance system through a description of the existing surveillance system and data before and after the revision.

**METHODS:**

After a historical review of surveillance system in Japan, the current surveillance system is described. Data sets of actual case numbers reported and incidence rate per 1,000,000 population are compared before and after the revision.

**RESULTS:**

Comparison of the data between the 2 periods revealed that most of the diseases have had declining trends after the new law was enacted with several exceptions. However, although no major break in continuity is observed in seriously perceived disease, in milder diseases there are striking gaps between the numbers reported in the mandatory and sentinel reporting framework. Sentinel reporting framework maintained the continuity of data without major gaps.

**CONCLUSIONS:**

From this perspective, the new surveillance system with two different frameworks of mandatory reporting for severe diseases and sentinel reporting for milder diseases seems to be working well. But continuous efforts should be made for evaluation and improvement of surveillance system and risk communication through the research on data analysis and effective communication method.

Public health surveillance is defined by the World Health Organization (WHO) as the "Systematic ongoing collection, collation, and analysis of data and the timely dissemination of information to those who need to know so that action can be taken." It is one of the essential components for infectious disease control and no doubt a starting point for them. Timely dissemination would be a key to effective surveillance. Unfortunately in Japan, however, although disease notification triggered the local response, data reported under several notification mechanisms had not been appropriately fedback to medical society and the public for many years. This leads to a decline in the motivation to report disease. In the meantime, circumstances of infectious diseases have been changing dramatically.^1^ Consequently, the infectious disease surveillance in Japan was no longer functioning as a tool for infectious disease control. In 1999, the Communicable Disease Prevention Law was completely revised, and the concept of "surveillance" was legally set forth and encouraged. In this paper, after historical review of infectious disease surveillance in Japan, the current surveillance system and data based on it are described to provide overview of the infectious disease surveillance system in Japan. Through these observations, the potential impact of the new surveillance system is discussed.

## HISTORICAL REVIEW OF INFECTIOUS DISEASE SURVEILLANCE IN JAPAN

Before 1999, the Communicable Disease Prevention Law which enacted in 1897 was the only legal framework for infectious disease control and gave a legal basis for 26 reportable diseases (Table 1). Legally defined communicable diseases (11) and specially designated communicable diseases (3) are expected to be reported immediately and notifiable communicable diseases (12) within 24 hours after diagnosis by medical practitioners to prefectural or designated/core city municipal public health authorities through local public heath centers. Sexually transmitted disease, Tuberculosis, and Acquired Immunodeficiency Syndrome (AIDS) have been covered by the Venereal Disease Prevention Law since 1948, the Tuberculosis prevention law since 1951 and the AIDS Prevention Law since 1989, respectively.

**Table 1.** Number of reported cases, mandatory reporting disease, Japan, 1993-1998.

	No. of reported cases						Incidence rate^*^

	1993	1994	1995	1996	1997	1998	
Legally defined communicable diseases^†^							
Cholera	92	90	306	40	89	61	0.90
Diphtheria	5	1	1	1	1	3	0.02
Dysentery	1,120	1,042	1,062	1,218	1,301	1,749	9.94
Meningococcal meningitis	7	6	3	4	5	6	0.04
Epidemic typhus	0	0	0	0	0	0	0.00
Japanese encephalitis	8	6	4	6	6	4	0.05
Paratyphoid fever	46	49	75	32	37	54	0.39
Plague	0	0	0	0	0	0	0.00
Scarlet fever	23	6	5	4	3	0	0.05
Smallpox	0	0	0	0	0	0	0.00
Typhoid fever	129	71	64	81	79	61	0.64

Specially designated communicable diseases^†^							
Acute poliomyelitis	3	1	1	0	0	0	0.01
Enterohemorrhagic *Escherichia coli* infection	…	…	…	1,287	1,941	2,077	14.08
Lassa fever	0	0	0	0	0	0	0.00

Notifiable communicable diseases^†^							
Anthrax	0	2	0	0	0	0	0.00
Filariasis	1	0	1	1	0	1	0.01
Infectious diarrhea	2	1	0	10	140	0	0.20
Influenza	16,655	2,404	22,393	8,774	8,816	14,778	97.98
Malaria	58	74	66	51	69	79	0.53
Measles	2,002	1,766	931	1,640	899	761	10.62
Pertussis	131	145	226	183	42	43	1.02
Rabies	0	0	0	0	0	0	0.00
Relapsing fever	0	0	0	0	0	0	0.00
Scrub typhus	712	652	529	423	487	538	4.43
Tetanus	33	44	45	44	47	47	0.35

Acquired Immunodeficiency Syndrome Prevention Law							
Acquired immunodeficiency syndrome							
Asymptomatic	277	298	277	376	397	422	2.72
Acquired immunodeficiency syndrome	86	136	169	234	250	231	1.47

Venereal Diseases Prevention Law							
Chancroid	9	4	5	6	3	4	0.04
Gonorrhea	1,724	1,448	1,699	2,201	2,355	3,096	16.62
Lymphogranuloma inguinale	1	0	0	1	1	1	0.01
Syphilis	804	666	530	565	448	553	4.73

…: Not applicable.

*: Incidence rate is per year per 1,000,000 population in 1993-1998

†: Defined by the Communicable Disease Prevention Law

In 1981 the national (sentinel) infectious disease surveillance program initiated consisting of (1) sentinel surveillance for 27 common infectious diseases (Table 7), and (2) infectious agents surveillance among local public health institutes, in order to fill the gap and vacuum of the national reportable diseases based on the law. This surveillance program, however, did not have any legal basis.

In April 1999, the Communicable Disease Prevention Law, the Venereal Disease Prevention Law, and the AIDS Prevention Law were abrogated, and the Law Concerning the Prevention of Infectious Diseases and Medical Care for Patients of Infections (hereafter referred to as the Infectious Disease Control Law) was enacted.^2^ In this new law, infectious disease surveillance was designated as one of the important components for disease control, and the sentinel surveillance was revised and incorporated as the national epidemiological surveillance for infectious disease (NESID) in combination with national notifiable diseases. In order to strengthen the surveillance system based on notification from physicians, collection, and analysis of the incidence and the trend of infectious diseases, effective and timely feedback of such information to the general public as well as those working in medical fields are proposed.

Infectious diseases included in this law were categorized into I through IV with specific means for control based upon the public health impact of each disease. The local outbreak of psittacosis and global outbreak of Severe Acute Respiratory Syndrome (SARS) proved that the law could not provide adequate measures against vectors and animals such as restriction of importing infecting animals, control of infected animals and extermination of vectors like mosquitoes and rats. Therefore in November 2003, the law was revised to create the new category IV including zoonotic and vector-borne diseases.^3^ The previous Category IV infectious diseases, except those included in the new Category IV, have been placed under the new Category V.

Finally, target diseases for the law were categorized and listed as shown in Table 2-4 and 8. These were re-organized by another revision in December 2006, but are not referred to in this paper.

**Table 2.** Number of reported cases of Category I, II and III, mandatory reporting disease, Japan, 1999-2005.^*^

	No. of reported cases							Incidence rate^‡^

	1999^†^	2000	2001	2002	2003	2004	2005	
Category I (all cases to be notified promptly after diagnosis)^*^								
Crimean-Congo hemorrhagic fever	0	0	0	0	0	0	0	0.00
Ebola hemorrhagic fever	0	0	0	0	0	0	0	0.00
Lassa fever	0	0	0	0	0	0	0	0.00 (0.00)
Marburg disease	0	0	0	0	0	0	0	0.00
Plague	0	0	0	0	0	0	0	0.00 (0.00)
Severe Acute Respiratory Syndrome (SARS)	…	…	…	…	0	0	0	0.00
Smallpox	…	…	…	…	0	0	0	0.00 (0.00)

Category II (all cases to be notified promptly after diagnosis)^*^								
Acute poliomyelitis	0	1	0	0	0	0	0	0.00 (0.01)
Cholera	39	58	50	51	25	86	56	0.43 (0.90)
Diphtheria	1	0	0	0	0	0	0	0.00 (0.02)
Paratyphoid fever	30	20	22	35	44	88	20	0.30 (0.39)
Shigellosis	620	843	844	699	473	594	553	5.24
Typhoid fever	72	86	65	63	62	67	50	0.51 (0.64)

Category III (all cases to be notified promptly after diagnosis)^*^								
Enterohemorrhagic *Escherichia coli* infection	3,117	3,642	4,435	3,183	2,999	3,715	3,589	28.20 (14.08)

…: Not applicable.

* : Defined by the Law Concerning the Prevention of Infectious Diseases and Medical Care for Patients of Infections

† : From April to December, 1999

‡ : Incidence rate is per year per 1,000,000 population in 2000-2005, and the rate in 1993-1998 is in parentheses

**Table 3.** Number of reported cases of Category IV, mandatory reporting disease, Japan, 1999-2005.^*^

	No. of reported cases							Incidence rate^‡^

	1999^†^	2000	2001	2002	2003	2004	2005	
Category IV (all cases to be notified promptly after diagnosis)^*^								
Anthrax	0	0	0	0	0	0	0	0.00 (0.00)
Avian influenza virus infection	…	…	…	…	0	0	0	0.00
Botulism	…	…	…	…	0	0	3	0.01
Brucellosis	0	0	0	1	0	0	2	0.00
Coccidioidomycosis	0	1	2	3	1	5	5	0.02
Dengue fever	9	18	50	52	32	49	74	0.36
Echinococcosis								
Granulosus	1	2	2	2	1	1	2	0.01
Multilocularis	6	20	13	8	19	25	18	0.13
Epidemic typhus	0	0	0	0	0	0	0	0.00 (0.00)
Hantavirus pulmonary syndrome	0	0	0	0	0	0	0	0.00
Hemorrhagic fever with renal syndrome	0	0	0	0	0	0	0	0.00
Hepatitis A	761	381	491	502	303	139	170	2.60
Hepatitis E	0	3	0	16	30	37	42	0.17
Herpes B virus infection	0	0	0	0	0	0	0	0.00
Japanese encephalitis	5	7	5	8	1	5	7	0.04 (0.05)
Japanese spotted fever	39	38	40	36	52	66	62	0.38
Legionellosis	56	154	86	167	146	161	281	1.30
Leptospirosis	…	…	…	…	1	18	17	0.09
Lyme disease	14	12	15	15	5	5	8	0.08
Lyssavirus infection	…	…	…	…	0	0	0	0.00
Malaria	112	154	109	83	78	75	67	0.74 (0.53)
Monkeypox	…	…	…	…	0	0	0	0.00
Nipah virus infection	…	…	…	…	0	0	0	0.00
Psittacosis	23	18	35	54	44	40	34	0.29
Q fever	12	24	42	47	9	7	8	0.18
Rabies	0	0	0	0	0	0	0	0.00 (0.00)
Relapsing fever	0	0	0	0	0	0	0	0.00
Scrub typhus (Tsutsugamushi disease)	556	791	491	338	402	313	345	3.51 (4.43)
Tularemia	…	…	…	…	0	0	0	0.00
West Nile fever	…	…	…	0	0	0	1	0.00
Yellow fever	0	0	0	0	0	0	0	0.00

…: Not applicable.

* : Defined by the Law Concerning the Prevention of Infectious Diseases and Medical Care for Patients of Infections

† : From April to December, 1999

‡ : Incidence rate is per year per 1,000,000 population in 2000-2005, and the rate in 1993-1998 is in parentheses

**Table 4.** Number of reported cases of Category Va, mandatory reporting disease, Japan, 1999-2005.^*^

	No. of reported cases							Incidence rate^‡^

	1999^†^	2000	2001	2002	2003	2004	2005	
Category Va (all cases to be reported by all physicians within 7days after diagnosis)^*^								
Acquired immunodeficiency syndrome (AIDS)								
Asymptomatic	346	413	570	547	564	699	753	4.64 (2.72)
AIDS	215	331	320	312	337	386	360	2.68 (1.47)
Other	27	50	57	57	69	77	90	0.52
Amebiasis	276	378	429	465	520	610	698	4.05
Acute encephalitis	…	…	…	…	12	166	188	0.48
Congenital rubella syndrome	0	1	1	1	1	10	2	0.02
Creutzfeldt-Jakob disease	92	108	133	147	118	175	152	1.09
Cryptosporidiosis	4	3	11	109	8	92	12	0.31
Giardiasis	42	98	137	113	103	94	86	0.83
Severe invasive streptococcal infections	22	47	47	92	53	52	60	0.46
Syphilis	751	759	585	575	509	533	543	4.58 (4.73)
Meningococcal meningitis	10	15	8	9	18	21	10	0.11 (0.04)
Tetanus	66	91	80	106	73	101	115	0.74 (0.35)
Vancomycin-resistant Enterococcus infection	23	36	40	44	59	58	69	0.40
Vancomycin-resistant Staphylococcus aureus infection	…	…	…	…	0	0	0	0.00
Viral hepatitis (excluding hepatitis A and E)								
Type B	510	425	330	332	245	241	208	2.33
Type C	136	119	65	61	65	43	57	0.54
Type D	0	0	0	0	0	0	0	0.00
Other	74	40	29	23	19	7	10	0.17
Unknown	36	22	14	14	4	2	1	0.07

…: Not applicable.

* : Defined by the Law Concerning the Prevention of Infectious Diseases and Medical Care for Patients of Infections

† : From April to December, 1999

‡ : Incidence rate is per year per 1,000,000 population in 2000-2005, and the rate in 1993-1998 is in parentheses

## DESCRIPTION OF CURRENT SURVEILLANCE SYSTEM

**Figure 1.**
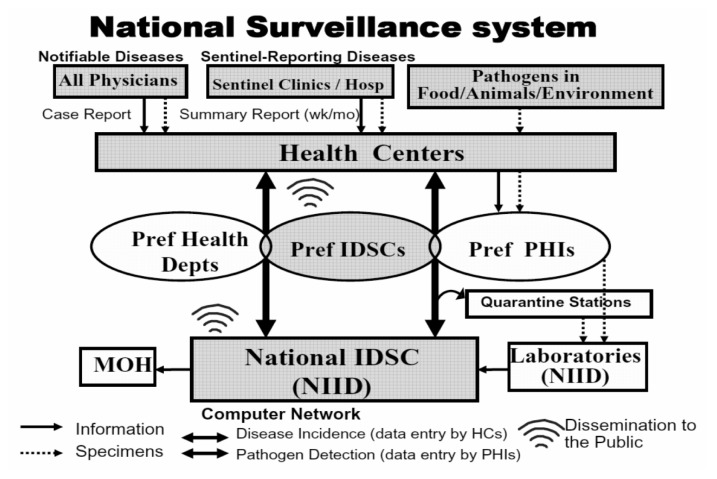
National surveillance structure in the national epidemiological surveillance for infectious disease(NESID).

Surveillance flow and function required at each level are shown in the Figure 1. All physicians must report diseases of Categories I-IV immediately and Va within 7 days after identification to local public health centers which are the primary level institution for disease control and prevention strategically located all over the nation. Category Vb disease, which includes sentinel reporting diseases, should be reported by designated sentinel medical institutions weekly or monthly with the number of clinical cases aggregated by sex and age groups. All reports should be compatible with the reporting criteria which were documented in detail for each disease including clinical and laboratory case definitions for categories I-Va and Vb of hospital sentinel reporting disease, and only clinical case definitions for other Vb sentinel reporting diseases.^4^

Local public health centers are expected to enter data into the nationwide electronic surveillance system, which enables data to be shared throughout the system. All reports are analyzed, interpreted and published by various forms of tabulations and graphs with commentary text weekly or monthly at intermediate and national level infectious disease surveillance centers, as stipulated by law. However, since category Vb diseases are reported by designated sentinel medical institutions, not by all physicians, data are displayed by weekly reported number per sentinel so as to provide understanding of the epidemic situation and disease trend. In order to be consistently representative, sentinels are expected to be selected as randomly as possible, and the numbers of sentinels per public health center coverage area are determined in proportion to the population of the area.^5^

## SUMMARY OF REPORTS FROM SURVEILLANCE SYSTEM

In order to understand the reporting situation under the current surveillance system, all the reports after the enactment of the new law were summarized in this section. To illustrate surveillance under the new law, data are compared with those under the old law.

### Materials and Methods

Surveillance data in 1999-2005 based on the Infectious Disease Control Law were derived from the annual report of NESID. To compare the situation of surveillance, 6-year data sets in 1993-1998 based on the Communicable Disease Prevention Law are obtained from the Statistics on communicable diseases. Data for syphilis and AIDS are obtained from the statistics based on the report on the Venereal Disease Prevention Law and the annual report for the AIDS Prevention Law, respectively. Data in 1999 are only available in April to December because of the change of the law in April 1999. Finally, data sets of actual case numbers reported and incidence rate per 1,000,000 population are compared before and after the revision. Incidence rates of 1993-1998 are calculated using 1995 census population and those of 1999-2005 are based on the 2002 population.

In order to discuss the value of sentinel surveillance, sentinel surveillance data in 1993-1998 are extracted from the annual report of the national infectious disease surveillance program and compared with the data in 1999-2005 from the annual report of NESID. Annual case numbers per sentinel, and number of sentinels are compared for the two periods. In addition, data are compared with cases reported under the old law, where data on the same diseases are available.

### Secular Trend in Target Infectious Diseases

Annual reported number and average incidence rate per 1,000,000 population through 6 years of all notifiable diseases under the old law are shown in Table 1, and data on diseases under the new law in Table 2-4. Data are compared between two periods whenever data on the relevant disease entity are available. Most of the diseases have declining trends in the new law period compared with before, although occasional increases are observed in paratyphoid fever and cholera. Exceptionally, enterohemorrhagic E-coli infection, malaria, tetanus and AIDS showed more cases under the new law. Cases with dysentery decreased under the new law, but dysentery in the old era included both disease entities caused by Shigella dysentery and entamoeba histolytica.

As to the sentinel reporting diseases, the number of sentinels under the new law increased mainly because of the change in selection criteria (Table 5, 6). Influenza sentinels in the old period are the same as pediatric ones, but are expanded in combination with internal medicine clinics mainly for adult patients under the new law. Ophthalmologic and sexually transmitted diseases sentinels increased in number, but hospital sentinels remain in the same level as no major change on selection criteria.

**Table 5.** Number of sentinel medical institutions, Japan, 1993-1998.

Sentinels^*^	1993	1994	1995	1996	1997	1998
Pediatric disease	2,425	2,425	2,440	2,412	2,412	2,412
Ophthalmologic disease	315	314	314	316	316	316
Sexually transmitted disease	596	599	604	606	606	606
Hospital	544	540	521	518	518	518

* : Based on the national infectious disease surveillance program

**Table 6.** Number of sentinel medical institutions, Japan, 1999-2005.

Sentinels^*^	1999†	2000	2001	2002	2003	2004	2005
Influenza	4,128	4,585	4,649	4,698	4,703	4,653	4,729
Pediatric disease	2,875	2,978	3,019	3,036	3,041	3,019	3,065
Ophthalmologic disease	589	625	634	634	634	633	649
Sexually transmitted disease	855	897	911	917	920	916	931
Hospital Weekly	456	459	470	473	471	475	471
Monthly	445	457	458	465	468	468	471

* : Defined by the Law Concerning the Prevention of Infectious Diseases and Medical Care for Patients of Infections

† : From April to December, 1999

**Table 7.** Number of reported cases per sentinel, sentinel reporting disease, Japan, 1993-1998.

	No. of reported cases per sentinel						Average^*^

	1993	1994	1995	1996	1997	1998	
Pediatric disease sentinel (weekly report)^†^							
Atypical pneumonia	10.95	8.90	9.53	11.09	9.22	8.46	9.69
Chickenpox	77.01	73.36	76.23	78.79	76.75	67.02	74.86
Erythema infectiosum	7.02	5.29	5.69	15.62	22.65	12.72	11.50
Exanthem subitum	35.73	36.76	35.02	34.69	35.66	34.92	35.46
Group A streptococcal pharyngitis	29.02	32.98	24.55	31.00	34.77	33.94	31.04
Hand, foot and mouth disease	38.15	22.59	65.04	10.17	30.98	52.06	36.50
Herpangina	29.87	36.94	32.61	40.03	36.25	32.62	34.72
Infectious gastroenteritis	169.23	165.97	193.20	162.71	173.88	161.40	171.07
Infantile vomiting and diarrhea	36.29	32.48	47.18	31.43	34.96	33.19	35.92
Influenza	262.49	44.77	310.14	146.27	162.91	235.39	193.66
Measles (excluding adult)	14.25	8.89	7.32	9.55	6.49	4.05	8.42
Mumps	38.04	52.65	29.05	46.72	62.22	56.60	47.55
Pertussis	1.51	1.85	2.32	2.36	1.13	0.98	1.69
Pharyngoconjunctival fever	3.85	1.76	4.40	3.43	2.44	2.30	5.09
Rubella	60.97	14.79	6.67	11.10	19.57	9.14	20.37
Kawasaki disease (Clinic sentinel)	0.46	0.57	0.55	0.56	0.59	0.57	0.55

Ophthalmologic disease sentinel (weekly report)^†^							
Acute hemorrhagic conjunctivitis	5.67	20.25	3.68	1.39	1.28	1.54	5.63
Epidemic keratoconjunctivitis	41.93	53.19	70.36	63.31	59.84	53.88	57.08
Pharyngoconjunctival fever	2.03	2.54	2.49	2.35	2.15	2.79	2.39

Sexually transmitted disease sentinel (monthly report)^†^							
Condyloma acuminatum	4.75	4.02	3.55	3.39	3.42	3.81	3.83
Genital chlamydial infection	23.13	23.91	22.74	23.80	25.97	28.50	24.68
Genital herpes	9.65	9.82	9.44	10.15	9.81	9.43	9.72
Gonorrhea	11.28	10.48	11.06	13.04	14.19	16.36	12.73
Syphilis	…	…	…	…	1.46	1.46	
Trichomoniasis	6.52	6.12	4.99	4.51	3.93	3.77	4.97

Sentinel hospital (monthly report)^†^							
Aseptic meningitis	3.96	5.11	3.11	2.96	6.42	9.86	5.24
Bacterial meningitis	0.42	0.44	0.47	0.50	0.55	0.46	0.48
Encephalitis/Encephalopathy/Myelitis/ Reye syndrome	0.44	0.35	0.35	0.35	0.39	0.48	0.39
Hepatitis A	1.47	1.32	0.67	0.67	0.49	0.26	0.81
Hepatitis B	1.24	0.99	0.80	1.02	0.88	0.89	0.97
Hepatitis C	…	…	…	…	1.74	1.74	
Other hepatitis	4.88	2.86	2.23	2.68	2.84	1.62	2.85
Kawasaki disease (Hospital sentinels)	2.89	3.30	3.52	3.27	3.32	3.52	3.30

…: Not applicable.

* : Average of the numbers of reported cases per sentinel in 1993-1998

† : Based on the national infectious disease surveillance program

**Table 8.** Number of reported cases per sentinel, sentinel reporting disease, Japan, 1999-2005.

	No. of reported cases per sentinel							Average^*^	

	1999^†^	2000	2001	2002	2003	2004	2005		
Influenza sentinel (weekly report)^‡^									
Influenza	15.32	167.93	65.70	159.01	247.14	165.50	330.65	189.32	(193.66)

Pediatric disease sentinel (weekly report)^‡^									
Chickenpox	56.50	92.36	89.90	86.73	82.39	81.46	79.05	85.32	(74.86)
Erythema infectiosum	6.47	11.50	22.41	19.02	11.77	16.20	12.82	15.62	(11.50)
Exanthem subitum	33.30	42.57	41.48	38.43	38.39	37.53	34.72	38.85	(35.46)
Group A streptococcal pharyngitis	31.40	53.10	51.32	51.38	54.77	68.58	60.27	56.57	(31.04)
Hand, foot and mouth disease	17.67	68.96	42.32	29.98	56.78	29.39	28.84	42.71	(36.50)
Herpangina	53.84	49.45	46.44	36.71	48.89	34.94	47.07	43.92	(34.72)
Infectious gastroenteritis	176.55	297.57	289.58	293.12	298.19	315.56	307.32	300.22	(171.07)
Measles (excluding adult)	2.04	7.57	11.20	4.11	2.72	0.51	0.18	4.38	(8.42)
Mumps	24.02	44.62	84.37	59.56	27.86	42.26	61.28	53.33	(47.55)
Pertussis	0.92	1.28	0.58	0.48	0.51	0.73	0.44	0.67	(1.69)
Pharyngoconjunctival fever	3.73	6.81	8.49	5.11	13.40	20.23	16.29	11.72	(5.09)
Respiratory syncytial virus infection	…	…	…	…	§	§	§	§	
Rubella	1.03	1.05	0.85	0.98	0.92	1.40	0.29	0.92	(20.37)

Ophthalmologic disease sentinel (weekly report)^‡^									
Acute hemorrhagic conjunctivitis	1.84	2.29	2.11	1.60	1.61	1.21	1.12	1.66	(5.63)
Epidemic keratoconjunctivitis	40.65	65.40	62.30	54.53	48.51	44.02	45.78	53.42	(57.08)

Sexually transmitted disease sentinel (monthly report)^‡^									
Condyloma acuminatum	3.73	5.08	5.68	6.22	6.80	7.17	7.30	6.38	(3.83)
Genital chlamydial infection	29.28	41.28	44.83	47.73	45.59	41.65	37.66	43.12	(24.68)
Genital herpes	7.68	9.97	10.22	10.54	10.69	10.67	11.02	10.52	(9.72)
Gonorrhea	13.86	18.87	22.68	23.91	22.50	19.02	16.11	20.52	(12.73)

Sentinel hospital (weekly report)^‡^									
Acute encephalitis (excluding Japanese encephalitis and West Nile encephalitis)	0.28	0.32	0.29	0.23	0.21	…	…	0.26	
Aseptic meningitis	2.47	4.08	2.67	6.31	3.45	2.16	1.64	3.39	(5.24)
Bacterial meningitis	0.52	0.56	0.59	0.63	0.63	0.80	0.66	0.65	(0.48)
Chlamydial pneumonia (excluding psittacosis)	0.28	0.39	0.39	0.52	0.43	0.51	0.68	0.49	
Measles in adults	0.18	0.93	1.98	0.93	0.98	0.12	0.01	0.83	
Mycoplasmal pneumonia	2.49	4.55	9.07	9.05	12.08	12.66	15.03	10.41	

Sentinel hospital (monthly report)^‡^									
Methicillin-resistant Staphylococcus aureus infection	24.92	39.42	40.19	43.47	45.52	46.64	48.01	43.88	
Multi-drug-resistant Pseudomonas aeruginosa infection	0.98	1.21	1.33	1.54	1.62	1.43	1.48	1.44	
Penicillin-resistant Streptococcus pneumoniae infection	4.78	9.46	11.47	13.19	13.78	14.30	13.23	12.57	

…: Not applicable.

* : Average of the numbers of reported cases per sentinel in 2000-2005, and average in 1993-1998 in parentheses

† : Defined by the Law Concerning the Prevention of Infectious Diseases and Medical Care for Patients of Infections

§ : The cases were reported, but the number of cases per sentinel was not calculated.

Under such circumstances, many sentinel reporting diseases increased in terms of the number of reports per sentinel, although they fluctuated in epidemicity (Table 7, 8). But several vaccine preventable diseases including measles, rubella and pertussis decreased dramatically in the last 7 years. Continuity of the reported number is considered to be maintained throughout the observed period.

Influenza, measles, pertussis, scarlet fever and infectious diarrhea were mandatory notifiable diseases in the old period (Table 1), but they were monitored by the national sentinel surveillance program as well since 1981 (Table 7, 8). Comparison reveals a far greater number of reported cases in the sentinel system than in the mandatory system. This might be partly caused by the difference in case definition. Reports of influenza and infectious diarrhea in the mandatory system are based on clinical characteristics in the same manner as in the sentinel system; and reports of measles, pertussis and scarlet fever based on the clinical diagnosis were also acceptable in the mandatory system. Since there were no documented reporting criteria under the old law, reports mainly depended upon the clinician's decision irrespective of whether or not laboratory confirmation was made. Similar observations were made for syphilis, where there were 887 reports from 606 sentinels, but only 553 cases were reported from all physicians in 1998.

## DISCUSSION

The Communicable Disease Prevention Law enacted in 1st April 1898 long provided the legal framework for infectious disease control in Japan. Mandatory reporting of national notifiable disease based upon this law was the only system for infectious disease surveillance. The basic policy of the law was the traditional attempt to prevent the massive expansion of infectious disease by disease notification and following isolation and quarantine. In the 1990's, however, the circumstances surrounding infectious diseases showed drastic changes including emerging and re-emerging infectious diseases, globalization of travel and trade, animal diseases crossing into human populations, and accidental or deliberate release of biological agents. These situations made an effective outbreak response more difficult. And lacking appropriate risk communication including effective feedback of infectious disease information, clinicians were discouraged from reporting disease in compliance with the Communicable Disease Prevention Law.

In such circumstances, the Infectious Disease Control Law came into force as a means to emphasize the promotion of infectious disease prevention for society as a whole. As part of the efforts in such a shift, the new law underscored the importance of surveillance and provided the public and medical professionals with the information necessary to prevent infectious diseases based upon data from surveillance.

In the present study we describe the surveillance system and summarize the data reported before and after the overall reform of the surveillance system. An attempt was made to envisage the potential impact of the new surveillance system on disease reporting by comparing data under the new law with data under the old one.

No drastic changes, in other words, no break in continuity was observed in most of the diseases which listed in notifiable diseases in both periods, at least, in the diseases which were perceived as serious among the community in those days. Several reports have indicated that reporting completeness in the disease surveillance varied from 9% to 99% and was most strongly associated with the disease being reported. This may be related to the perceived seriousness of these diseases or to the greater financial and human resources devoted to treating and preventing them.^6^^,^^7^ Consistent with these observations, most of the notifiable diseases showed a similar incidence rate without any discontinuity of the data between the two periods.

Striking observations are found in the diseases which are perceived to be milder and non-life-threatening. Considerable gaps were noted for influenza, measles, pertussis and infectious diarrhea between the notifiable and sentinel reporting framework. As there were no major gaps throughout the overall observed period of the sentinel reporting framework, it is considered that data from sentinel reporting framework reflect the real situation more than those from the mandatory system. Although scarlet fever is not necessarily the same with group A streptococcal infection, it is curable by antibiotics and no longer life-threatening disease. Therefore, its surveillance is better on a sentinel system. Although no documented case definition was available under the old law, clinicians might have had a different perception of reporting criteria in these two systems.

One of the noteworthy features of the new surveillance system were disease categorization according to the disease impact and surveillance was correspondingly re-organized with the two different frameworks of mandatory and sentinel reporting. Through our current summary, the new surveillance system in combination with the mandatory system for severe diseases and sentinel system for milder diseases seems to be working better.

Although the characteristics of sentinels seem to have changed between the two periods, case reports per sentinels increased after the new law took effect. Exanthema subitum which is considered to be a standard disease to estimate the capture rate by existing sentinel clinics increased in the number of cases per sentinel. This might reflect the increased capture rate from re-designing the sentinel surveillance. But one must recall the increased general awareness of infectious diseases these days.

For most Japanese people, information about infectious diseases is not very familiar and sometimes difficult to understand. The index used in the sentinel system, the reported cases per sentinel, is not easy to understand. There have been several efforts to translate these data into more understandable expressions, and some of them are employed in the national system.^8^^-^^11^

It is notable that the new law has clearly stated the importance of dissemination of information for determining the appropriate action to be taken. According to the new law, the national and prefectural/municipal infectious disease surveillance centers have been organized so that these institutions can play a central role in implementing surveillance and information dissemination. Many infectious disease surveillance centers including national one publish infectious disease reports weekly, monthly, and/or when necessary on the web and in document form. In addition to these regular reports, papers in the academic field serve to facilitate the effective feedback of information.^12^^,^^13^

The new law classifies target diseases by their health impact, and it appears to improve the overall surveillance performance as the purpose of surveillance becomes clearer, especially for disease perceived to be milder. But since a single surveillance system obviously can not satisfy all the needs for a wide range of infectious disease control activities, disease-specific analysis should be made for further evaluation of the surveillance system and tailoring more specific surveillance to specific objectives.

In conclusion, under the new law different surveillance schemes have been developed suitable to assess disease impact with documented reporting criteria along with the development of systematic information dissemination systems. But continuous efforts are warranted for evaluation and further improvement of the surveillance system and risk communication through ongoing research on data analysis and effective communication methods.
